# Heme oxygenase-1 inducer and carbon monoxide–releasing molecule enhance the effects of gabapentinoids by modulating glial activation during neuropathic pain in mice

**DOI:** 10.1097/PR9.0000000000000677

**Published:** 2018-08-06

**Authors:** Kohei Godai, Yuichi Kanmura

**Affiliations:** Department of Anesthesiology and Critical Care Medicine, Graduate School of Medical and Dental Sciences, Kagoshima University, Kagoshima, Japan

**Keywords:** Gabapentinoids, Heme oxygenase-1, Spared nerve injury

## Abstract

**Introduction:**

Neuropathic pain is one of the most difficult-to-treat symptoms. Although gabapentinoids are classified as first-line drugs, they have only modest efficacy.

**Objectives:**

The aim of this study was to investigate whether treatment with the heme oxygenase-1 (HO-1) inducer cobalt protoporphyrin IX (CoPP) or the carbon monoxide–releasing molecule tricarbonyldichlororuthenium (II) dimer (CORM-2) can enhance the antinociceptive effects produced by gabapentinoids in mice with neuropathic pain.

**Methods:**

Neuropathic pain was induced by spared nerve injury (SNI) of the sciatic nerve. The mechanical threshold was tested using von Frey filaments. The expression of spinal HO-1, HO-2, the Ca^2+^ channel α_2_δ_1_ subunit, microglial markers, and M1 or M2 microglial markers was examined using reverse transcription polymerase chain reaction.

**Results:**

Treatment with CoPP or CORM-2 alleviated mechanical allodynia induced by SNI. CoPP or CORM-2 enhanced the antiallodynic effects of gabapentinoids (pregabalin or gabapentin) during SNI-induced mechanical allodynia. HO-1 inhibitor tin protoporphyrin IX (SnPP) prevented the antiallodynic effects of gabapentinoids (pregabalin or gabapentin) during SNI-induced mechanical allodynia. CoPP or CORM-2 increased HO-1 and Ca^2+^ channel α_2_δ_1_ subunit gene expression and the decreased gene expression of microglial markers, M1 microglial marker, or tumor necrosis factor in the ipsilateral spinal dorsal horn of mice with SNI. SnPP prevented HO-1 induction and glial inhibition, which were produced by gabapentinoids during SNI-induced mechanical allodynia.

**Conclusions:**

This study suggests that HO-1 plays crucial roles in the antiallodynic effects of gabapentinoids. Gabapentinoids attenuate the glial activation induced by SNI and some of these effects are mediated by HO-1.

## 1. Introduction

Neuropathic pain is a chronic debilitating condition caused by a lesion or disease affecting the somatosensory nervous system. Neuropathic pain is estimated to affect 3% to 17% of the general population.^39^ Gabapentinoids (pregabalin and gabapentin) are calcium channel α_2_δ ligands, which are considered to reduce central sensitization through the calcium channel α_2_δ subunit.^28,29^ Although gabapentinoids are classified as first-line drugs for neuropathic pain, they have only modest efficacy. The reported number needed to treat for 50% pain relief is relatively high among gabapentinoids. Numbers needed to treat are 7.7 for pregabalin and 7.2 for gabapentin.^12^ Neuropathic pain is one of the most difficult-to-treat symptoms and induces a high disease burden in affected patients.^2^ Increasing the efficacy of drug treatment is one strategy for improving the management of patients with neuropathic pain.

Heme oxygenase (HO) is the rate-limiting enzyme in the oxidative degradation of heme into carbon monoxide (CO), bilirubin, and iron. Carbon monoxide has anti-inflammatory properties.^36^ Two distinct isoforms of HO exist: HO-1 and HO-2. HO-1 is induced by various stresses, including oxidative stress and ischemia reperfusion stress.^11,13^ The HO-1 inducer cobalt protoporphyrin IX (CoPP) and the CO-releasing molecule tricarbonyldichlororuthenium (II) dimer (CORM-2) have been shown to enhance the analgesic effects of opioid analgesics in neuropathic pain.^6–8^ However, the effects of HO-1 on the analgesic effects of gabapentinoids are unknown.

Neuroimmune interactions play crucial roles in the development of pain hypersensitivity.^38^ In particular, macrophages/microglia derived from circulating monocytes are activated in the early course of acute inflammation and promote the development of hyperalgesia.^1,10^ Two distinct polarized phenotypes are referred to as classically activated (M1) and alternatively activated (M2) macrophages/microglia.^15,23^ M1 macrophages/microglia produce proinflammatory cytokines such as tumor necrosis factor (TNFα), interleukin-1β (IL-1β), or inducible nitric oxide synthase (iNOS), and lead to increased reactive oxygen species production. M2 macrophages/microglia produce anti-inflammatory cytokines and play a vital role in resolution of inflammation and tissue healing. Previous studies have shown that HO-1 signaling promotes macrophage/microglia polarization toward M2.^14,17,18^ Although HO-1 induction has been reported to decrease microglial activation in neuropathic pain models, the association between HO-1 signaling and microglial polarization is unknown.^19,20,32^

In this study, we investigated (1) the antiallodynic effects of CoPP or CORM-2 in spared nerve injury (SNI)-induced neuropathic pain, (2) the effects of coadministration of CoPP or CORM-2 with gabapentinoids on SNI-induced mechanical allodynia, and (3) the effects of CoPP or CORM-2 on the expression of α_2_δ_1_, M1/M2 microglial markers, or other genes.

## 2. Methods

### 2.1. Animals

Male C57BL6 mice aged 8 to 10 weeks were obtained from Japan SLC, Inc (Hamamatsu, Japan). The Animal Research Committee of Kagoshima University approved all experimental procedures, which were implemented according to the guidelines of the National Institutes of Health and the International Association for the Study of Pain.^44^ The mice were housed in groups of 4 or 5 per cage in a 12-hour light–dark cycle.

### 2.2. Neuropathic pain model

Neuropathic pain was induced by SNI of the sciatic nerve.^5^ The mice were deeply anesthetized by inhalation of 1.5% to 2.0% isoflurane (Abbott, Tokyo, Japan) through a nose cone. An incision was made at the midthigh level, and a section was made through the biceps femoris. The tibial and common peroneal nerves were ligated and transected together using 6-0 silk. A 1- to 2-mm section of the 2 nerves was removed. We carefully avoided any nerve damage to the sural nerve. The muscle and skin were closed with two 6-0 silk sutures.

### 2.3. Mechanical threshold

The mechanical threshold was determined using calibrated von Frey filaments (0.008–2.0 g, Aesthesio Precise Tactile Sensory Evaluator; Danmic Global, San Jose, CA) introduced serially to the hind paw in ascending order of strength, and the animals were placed in nontransparent plastic cubicles on a mesh floor for an acclimatization period of at least 30 minutes on the morning of the test day. A positive response was defined as rapid withdrawal and/or licking of the paw immediately on application of the stimulus. Filaments were tested 5 times per paw, and the paw withdrawal threshold was defined as the filament for which 3 or more withdrawals out of the 5 trials were observed.^25^ The person who conducted the tests was blinded to the treatments. The antinociception induced by the experimental drugs was expressed as the percentage of the maximal possible effect calculated according to the following equation:

### 2.4. Reverse transcription polymerase chain reaction

Because SNI mainly affects the L3–4 spinal neurons, we selected the L3–4 spinal cord level to study.^26^ Under deep anesthesia, the ipsilateral L3–4 spinal dorsal horn was rapidly removed, frozen on dry ice, and stored at −80°C. Total RNA of the spinal dorsal horn was extracted from the hind paw using Sepasol reagent (Nacalai Tesque, Kyoto, Japan). The synthesis of first-strand cDNA was performed using a High-Capacity RNA-to-cDNA Kit (Applied Biosystems, Carlsbad, CA) according to the manufacturer's instructions. Quantitative PCR was performed on an ABI Prism StepOnePlus Real-Time PCR System (Applied Biosystems) using TaqMan Fast Advanced Master Mix (Applied Biosystems) according to the manufacturer's instructions. The primers of the target genes were Aif1 (assay ID, Mm00479862_g1; NCBI Reference Sequence, NM_019467.2), Arg1 (assay ID, Mm00475988_m1; NCBI Reference Sequence, NM_007482.3), Cacna2d1 (assay ID, Mm00486607_m1; NCBI Reference Sequence, NM_001110843.1), Cd68 (assay ID, Mm03047343_m1; NCBI Reference Sequence, NM_001291058.1), Gfap (assay ID, Mm01253033_m1; NCBI Reference Sequence, NM_001131020.1), Hmox1 (assay ID, Mm00516005_m1; NCBI Reference Sequence, NM_010442.2), Hmox2 (assay ID, Mm00468922_m1; NCBI Reference Sequence, NM_001136066.2), Il1b (assay ID, Mm00434228_m1; NCBI Reference Sequence, NM_008361.3), Itgam (assay ID, Mm00434455_m1; NCBI Reference Sequence, NM_001082960.1), Nos2 (assay ID, Mm00440502_m1; NCBI Reference Sequence, NM_010927.3), and Tnf (assay ID, Mm00443258_m1; NCBI Reference Sequence, NM_001278601.1). Target gene expression was normalized to glyceraldehyde 3-phosphate dehydrogenase.

### 2.5. Experimental protocol

In the first experiment, we assessed the expression of neuropathic pain using the mouse model of SNI. After baseline measurements, neuropathic pain was induced. The animals were tested once a week for 8 weeks. Sham-operated mice were used as controls (n = 7 animals per group).

In a second set of experiments, we investigated the mechanical antiallodynic effects produced by intraperitoneal administration of different doses of an HO-1 inducer (CoPP), CORM-2, or the HO-1 inhibitor tin protoporphyrin IX (SnPP) in animals with SNI on day 7 after surgery (n = 7–8 animals per group).

In a third set of experiments, we evaluated the mechanical antiallodynic effects produced by intraperitoneal administration of CoPP or CORM-2 combined with intraperitoneal administration of low doses of pregabalin or gabapentin and their respective vehicles in animals with SNI on day 7 after surgery (n = 6–7 animals per group).

In another set of experiments, we evaluated the mechanical antiallodynic effects produced by intraperitoneal administration of 10 mg/kg of SnPP combined with intraperitoneal administration of high doses of pregabalin or gabapentin and their respective vehicles in animals with SNI on day 7 after surgery (n = 6–7 animals per group). The doses of CoPP, CORM-2, and SnPP combined with pregabalin or gabapentin were selected as those that produced a relevant effect in accordance with other studies and with the dose–response performed in this study.^19,34,35^

In another set of experiments, we evaluated the effects of CoPP and CORM-2 treatments on the expression of HO-1, HO-2, iNOS, Ca^2+^ channel α_2_δ_1_ subunit, microglial markers (CD11b and ionized calcium-binding adapter molecule-1, [Iba-1]), an M1 microglial marker (CD68), an M2 microglial marker (Arg-1), proinflammatory cytokines (TNFα and IL-1β), and an astrocyte marker (glial fibrillary acidic protein [GFAP]) in the ipsilateral spinal dorsal horn of mice with SNI at 7 days after surgery using reverse transcription polymerase chain reaction. In these experiments, SNI-affected mice treated with vehicle were used as controls (n = 4–6 samples per group).

Finally, in another set of experiments, we evaluated the effects of gabapentinoids and SnPP treatments on the expression of aforementioned mRNAs in the ipsilateral spinal dorsal horn of mice with SNI at 7 days after surgery using reverse transcription polymerase chain reaction. In these experiments, SNI-affected mice treated with vehicle were used as controls (n = 4–5 samples per group).

### 2.6. Drugs

CORM-2, pregabalin, and gabapentin were purchased from Sigma-Aldrich (St. Louis, MO), and CoPP and SnPP were purchased from Enzo Life Sciences, Inc (Farmingdale, NY). CoPP, CORM-2, and SnPP were dissolved in dimethyl sulfoxide (1% solution in saline). Pregabalin and gabapentin were dissolved in saline solution (0.9% NaCl). All drugs were freshly prepared before use. CoPP and CORM-2 were intraperitoneally administered 4 hours before testing in a final volume of 10 mL/kg. SnPP, pregabalin, and gabapentin were intraperitoneally administered 1 hour before testing.

### 2.7. Statistical analysis

Data are expressed as mean ± SEM. In the scatter plot, bars indicate the mean and SEM. The statistical analysis was performed using GraphPad Prism 7.0 (GraphPad Software, La Jolla, CA). All comparisons were run as 2-tailed tests. The mechanical responses induced by SNI vs sham in the ipsilateral paws of mice were compared using repeated-measures 2-way analysis of variance (ANOVA) followed by the Bonferroni multiple comparison test. The effects produced by the experimental drugs and the gene expression in the ipsilateral spinal dorsal horn were compared using 1-way ANOVA followed by the Tukey multiple comparison test. A *P* value of <0.05 was considered statistically significant.

## 3. Results

### 3.1. Induction of neuropathic pain

**Figure 1.**
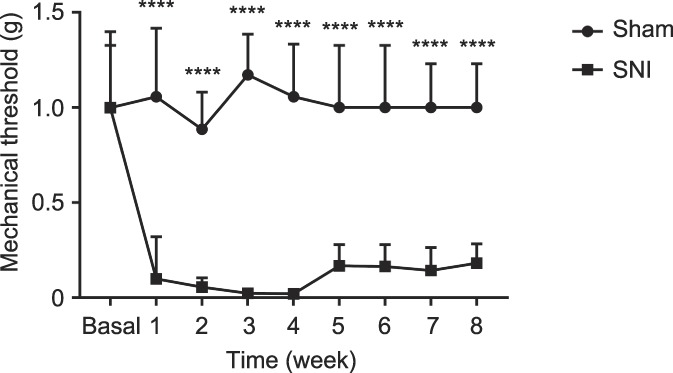
Time course of the mechanical threshold in the SNI neuropathic pain model. Mice with SNI developed a persistent mechanical allodynia of the ipsilateral hind paw compared with the sham-operated group (****P < 0.0001 vs Sham, 2-way ANOVA followed by the Bonferroni multiple comparison test, n = 7). ANOVA, analysis of variance; SNI, spared nerve injury.

Spared nerve injury produced ipsilateral mechanical allodynia 1 to 8 weeks after surgery (Fig. 1), as reported previously.^5^ For each test, 2-way ANOVA showed a significant effect of the surgery (*P* < 0.0001) and time (*P* < 0.0001) as well as their interaction (*P* < 0.0001).

### 3.2. Effects of CoPP, CORM-2, and SnPP on mechanical allodynia induced by spared nerve injury in mice

**Figure 2.**
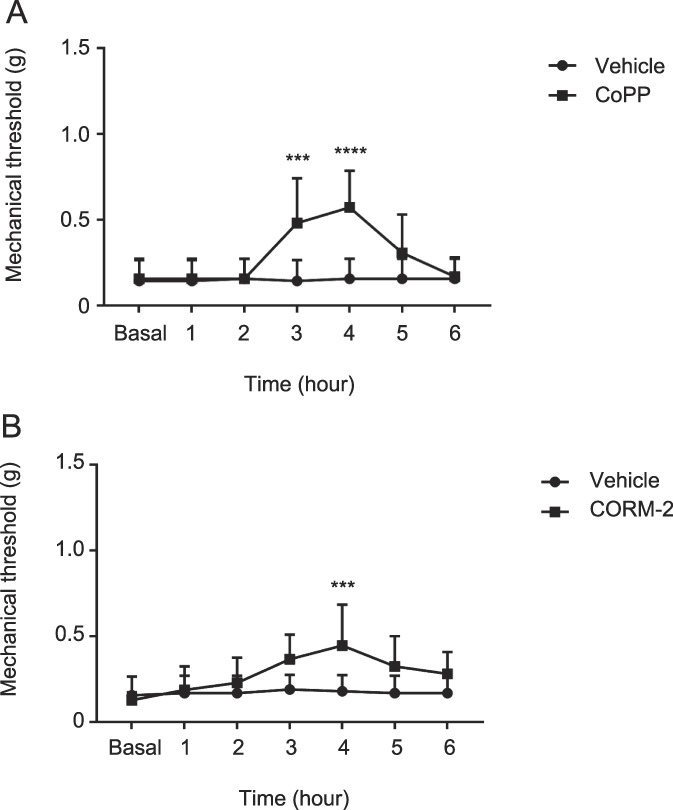
Time course of the antiallodynic effects of CoPP or CORM-2 during SNI neuropathic pain. Mice treated with CoPP (A) or CORM-2 (B) showed increased mechanical thresholds compared with the vehicle-treated mice (***P < 0.001 vs Vehicle, ****P < 0.0001 vs Vehicle, 2-way ANOVA followed by the Bonferroni multiple comparison test, n = 7, 8). ANOVA, analysis of variance; CoPP, cobalt protoporphyrin IX; CORM-2, carbon monoxide–releasing molecule tricarbonyldichlororuthenium (II) dimer; SNI, spared nerve injury.

We investigated the effects of intraperitoneal administration of different doses of CoPP (1, 3, and 10 mg/kg), CORM-2 (1, 3, and 10 mg/kg), and SnPP (3 and 10 mg/kg) on the mechanical allodynia induced by SNI at 7 days after surgery. Intraperitoneal administration of 3 and 10 mg/kg of CoPP or CORM-2 inhibited the mechanical allodynia induced by SNI (Table 1). There was no dose dependence on the antiallodynic effects of CoPP and CORM-2. The maximal antiallodynic effects were seen 4 hours after CoPP or CORM-2 injection (Fig. 2). By contrast, intraperitoneal administration of 3 or 10 mg/kg of SnPP did not alter the principal signs of neuropathic pain. Because 3 or 10 mg/kg of CoPP and CORM-2 produce a similar inhibitory effect, in the following experiments we used a dose of 3 mg/kg for both CoPP and CORM-2.

**Table 1:**
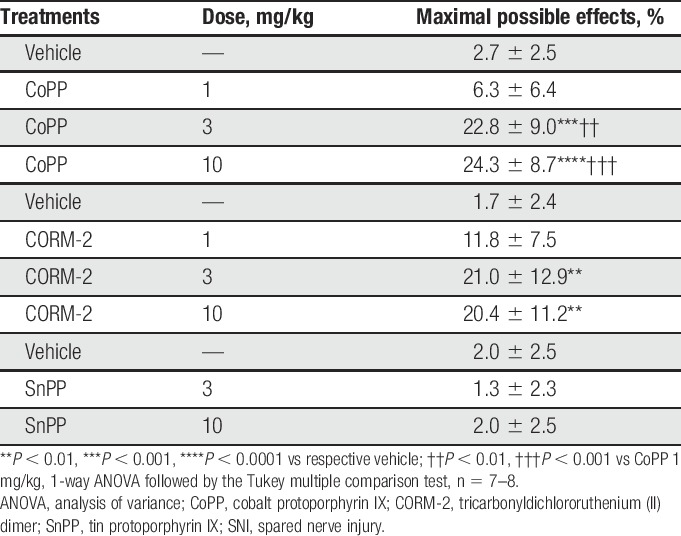
Effects of CoPP, CORM-2, and SnPP on the mechanical allodynia induced by SNI in mice.

### 3.3. Effects of CoPP and CORM-2 on antiallodynic responses to low doses of pregabalin and gabapentin

**Figure 3.**
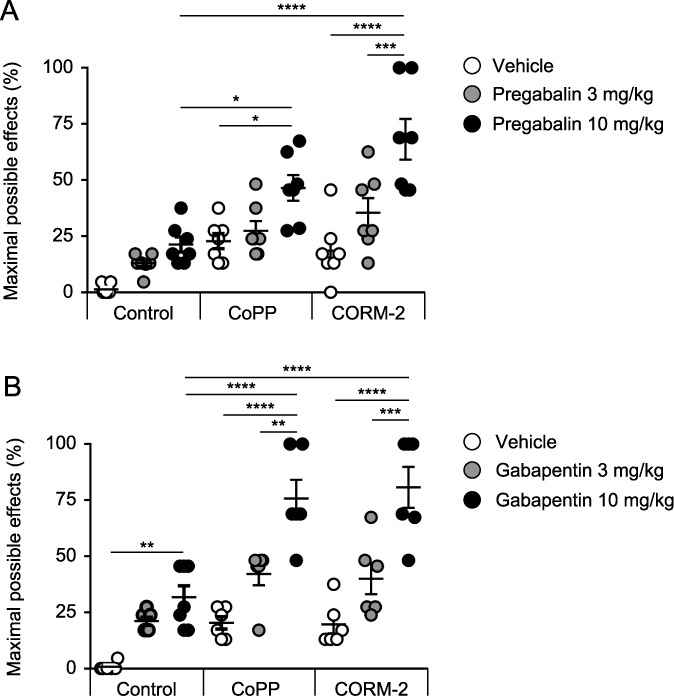
Effects of CoPP or CORM-2 treatment on the antiallodynic responses to low doses of pregabalin and gabapentin. Pretreatment with CoPP or CORM-2 significantly enhanced the attenuation of the mechanical antiallodynic effects of (A) pregabalin or (B) gabapentin compared with the control group, which was treated with vehicle (*P < 0.05, **P < 0.01, ***P < 0.001, ****P < 0.0001, 1-way ANOVA followed by the Tukey multiple comparison test, n = 6–7). Bars indicate the mean and SEM. ANOVA, analysis of variance; CoPP, cobalt protoporphyrin IX; CORM-2, tricarbonyldichlororuthenium (II) dimer.

We also investigated the effects of intraperitoneal administration of 3 mg/kg of CoPP and CORM-2 on the mechanical antiallodynic effects produced by intraperitoneal administration of low doses of pregabalin (3 and 10 mg/kg), gabapentin (3 and 10 mg/kg), or vehicle in mice with SNI at 7 days after surgery. Pretreatment with CoPP or CORM-2 significantly enhanced the attenuation of the mechanical antiallodynic effects of pregabalin compared with the control group, which was treated with vehicle (Fig. 3A). Pretreatment with CoPP or CORM-2 also significantly enhanced the attenuation of the mechanical antiallodynic effects of gabapentin compared with the control group (Fig. 3B).

### 3.4. Prevention of antinociceptive responses produced by high doses of pregabalin or gabapentin in spared nerve injury–affected mice with administration of the HO-1 inhibitor SnPP

**Figure 4.**
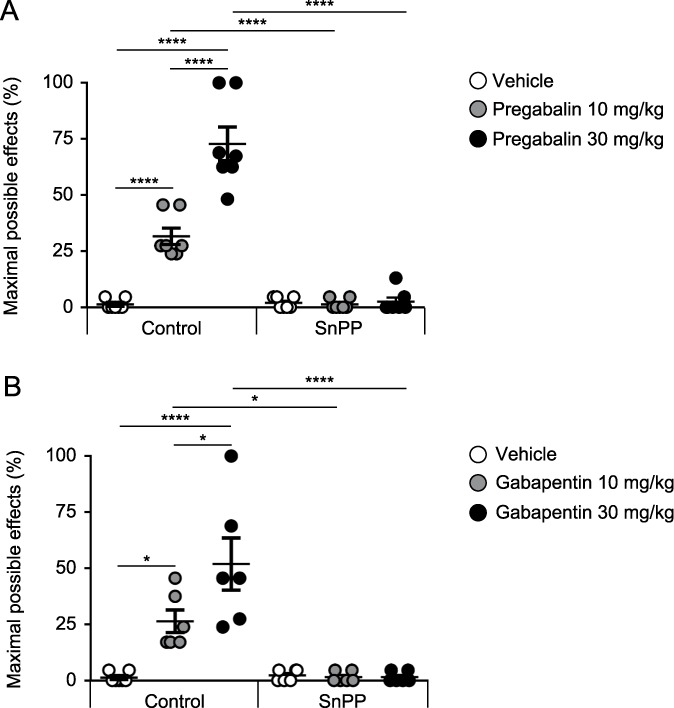
Reversal of the antinociceptive responses produced by high doses of pregabalin or gabapentin in SNI-affected mice with the administration of the HO-1 inhibitor SnPP. The coadministration of pregabalin or gabapentin with SnPP completely prevented the mechanical antiallodynic effects produced by (A) pregabalin or (B) gabapentin administered alone (*P < 0.05, ****P < 0.0001, 1-way ANOVA followed by the Tukey multiple comparison test, n = 6–7). Bars indicate the mean and SEM. ANOVA, analysis of variance; SnPP, tin protoporphyrin IX; SNI, spared nerve injury.

We assessed the effects of intraperitoneal administration of 10 mg/kg of SnPP or vehicle (DMSO 1%) on the mechanical antiallodynic effects produced by intraperitoneal administration of high doses of pregabalin (10 and 30 mg/kg) or gabapentin (10 and 30 mg/kg) in mice with SNI. The coadministration of pregabalin with SnPP completely prevented the mechanical antiallodynic effects produced by pregabalin administered alone (Fig. 4A). The coadministration of gabapentin with SnPP also completely prevented the mechanical antiallodynic effects produced by gabapentin administered alone (Fig. 4B).

### 3.5. Effects of CoPP and CORM-2 on HO-1, HO-2, α_2_δ_1_, and inducible nitric oxide synthase gene expression in the spinal dorsal horn of mice with spared nerve injury

**Figure 5.**
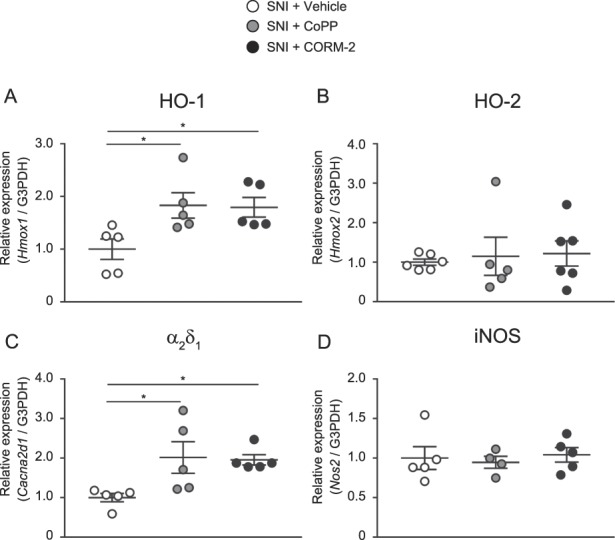
Effects of CoPP and CORM-2 on HO-1, HO-2, α_2_δ_1_, and iNOS gene expression in the spinal dorsal horn of mice with SNI. The expression of (A) HO-1 or (C) α_2_δ_1_ was significantly increased by CoPP or CORM-2 treatment. The mRNA level of (B) HO-2 or (D) iNOS in the ipsilateral spinal dorsal horn was not altered by CoPP or CORM-2 (*P < 0.05, 1-way ANOVA followed by the Tukey multiple comparison test, n = 4–6). Bars indicate the mean and SEM. ANOVA, analysis of variance; CoPP, cobalt protoporphyrin IX; CORM-2, tricarbonyldichlororuthenium (II) dimer; HO-1, heme oxygenase-1; HO-2, heme oxygenase-2; iNOS, inducible nitric oxide synthase; SNI, spared nerve injury.

The mRNA levels of HO-1, HO-2, α_2_δ_1_, and iNOS in the ipsilateral spinal dorsal horn from mice with SNI treated with vehicle, CoPP, or CORM-2 are shown in Figure 5. The expression of HO-1 (Fig. 5A) or α_2_δ_1_ (Fig. 5C) was significantly increased by treatment with CoPP or CORM-2. The mRNA level of HO-2 or iNOS in the ipsilateral spinal dorsal horn was not altered by CoPP or CORM-2 (Fig. 5B, D).

### 3.6. Effects of CoPP and CORM-2 on microglial or astrocytic markers and proinflammatory cytokine gene expression in the spinal dorsal horn of mice with spared nerve injury

**Figure 6.**
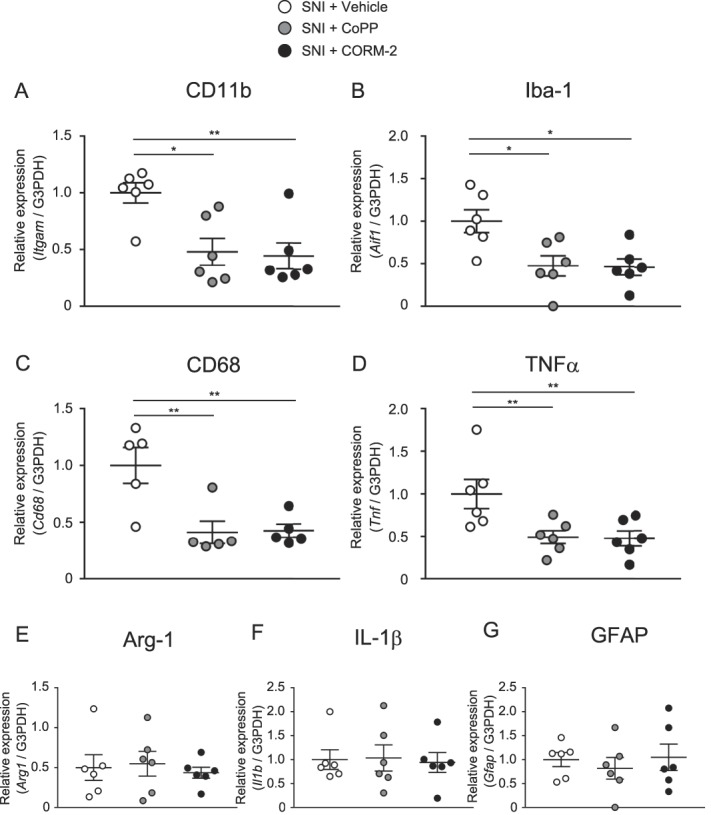
Effects of CoPP and CORM-2 on microglial or astrocytic markers or proinflammatory cytokine gene expression in the spinal dorsal horn of mice with SNI. (A–D) The expression of CD11b, Iba-1, CD68, or TNFα was significantly decreased by CoPP or CORM-2 treatment. (E–G) The mRNA level of arginase-1, IL-1β, or GFAP in the ipsilateral spinal dorsal horn was not altered by CoPP or CORM-2 (*P < 0.05, **P < 0.01, 1-way ANOVA followed by the Tukey multiple comparison test, n = 5–6). Bars indicate the mean and SEM. ANOVA, analysis of variance; Arg-1, arginase-1; CoPP, cobalt protoporphyrin IX; CORM-2, tricarbonyldichlororuthenium (II) dimer; GFAP, glial fibrillary acidic protein; Iba-1, ionized calcium-binding adapter molecule-1; IL-1β, interleukin-1β; SNI, spared nerve injury; TNFα, tumor necrosis factor.

Figure 6 shows the mRNA levels of CD11b or Iba-1 (microglial markers), GFAP (astrocyte marker), CD68 (M1 microglial marker), arginase-1 (M2 microglial marker), and TNFα or IL-1β (proinflammatory cytokines) in the ipsilateral spinal dorsal horn of mice with SNI treated with vehicle, CoPP, or CORM-2. The expression of CD11b, Iba-1, CD68, and TNFα (Fig. 6A–D) was significantly decreased by treatment with CoPP or CORM-2. The mRNA levels of arginase-1, IL-1β, and GFAP in the ipsilateral spinal dorsal horn were not altered by CoPP or CORM-2 (Fig. 6E–G).

### 3.7. Effects of gabapentinoids and SnPP on microglial or astrocytic markers and proinflammatory cytokine gene expression in the spinal dorsal horn of mice with spared nerve injury

**Figure 7.**
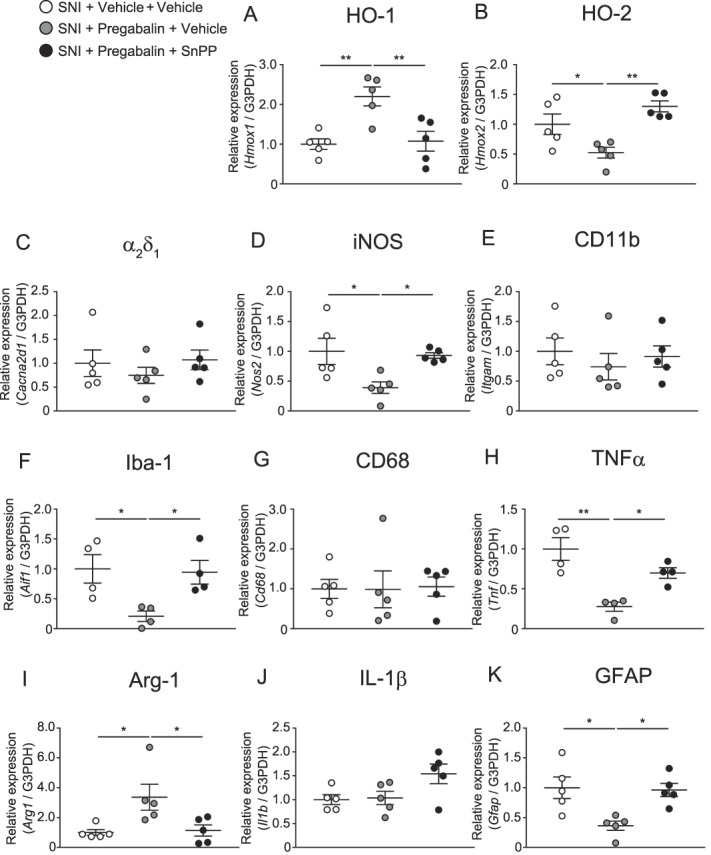
Effects of pregabalin alone or pregabalin combined with SnPP on HO-1, HO-2, α_2_δ_1_, iNOS, and microglial or astrocytic markers or proinflammatory cytokine gene expression in the spinal dorsal horn of mice with SNI (A–K) (*P < 0.05, **P < 0.01, 1-way ANOVA followed by the Tukey multiple comparison test, n = 4–5). Bars indicate the mean and SEM. ANOVA, analysis of variance; Arg-1, arginase-1; GFAP, glial fibrillary acidic protein; HO-1, heme oxygenase-1; HO-2, heme oxygenase-2; Iba-1, ionized calcium-binding adapter molecule-1; IL-1β, interleukin-1β; iNOS, inducible nitric oxide synthase; SNI, spared nerve injury; TNFα, tumor necrosis factor.

Figure 7 shows the mRNA levels of HO-1, HO-2, α_2_δ_1_, iNOS, CD11b, Iba-1, GFAP, CD68, arginase-1, TNFα, and IL-1β in the ipsilateral spinal dorsal horn of mice with SNI treated with vehicle, pregabalin alone, or pregabalin combined with SnPP. The increased expressions of HO-1 and Arg-1 and decreased expressions of HO-2, iNOS, Iba-1, TNF-α, and GFAP were detected in mice treated with pregabalin alone. The altered expression of the mRNAs was prevented by coadministration of SnPP.

**Figure 8.**
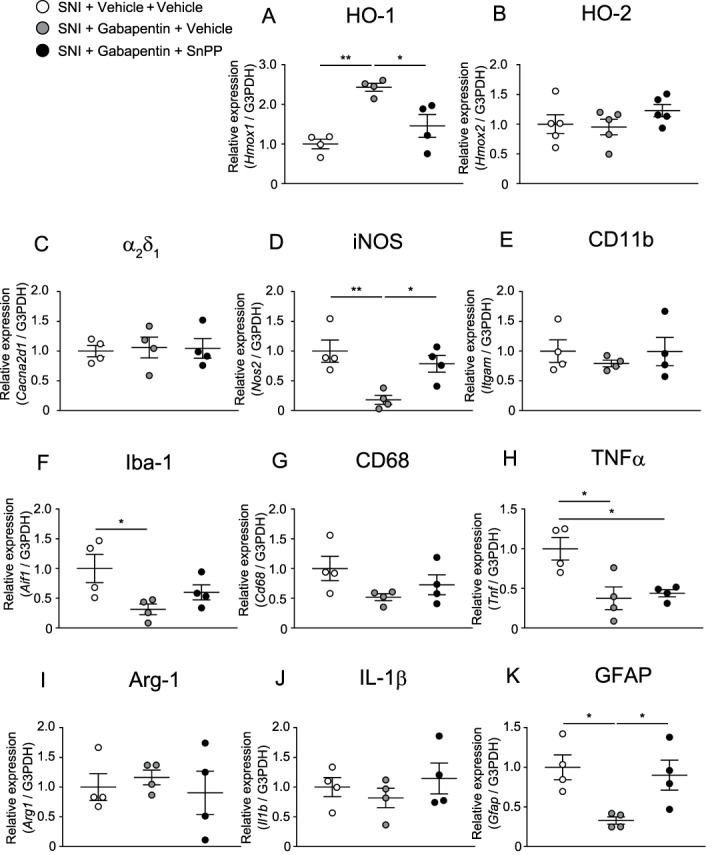
Effects of gabapentin alone or gabapentin combined with SnPP on HO-1, HO-2, α_2_δ_1_, iNOS, and microglial or astrocytic markers or proinflammatory cytokine gene expression in the spinal dorsal horn of mice with SNI (A–K) (*P < 0.05, **P < 0.01, 1-way ANOVA followed by the Tukey multiple comparison test, n = 4–5). Bars indicate the mean and SEM. ANOVA, analysis of variance; Arg-1, arginase-1; GFAP, glial fibrillary acidic protein; HO-1, heme oxygenase-1; HO-2, heme oxygenase-2; Iba-1, ionized calcium-binding adapter molecule-1; IL-1β, interleukin-1β; iNOS, inducible nitric oxide synthase; SNI, spared nerve injury; TNFα, tumor necrosis factor.

Figure 8 shows the mRNA levels of HO-1, HO-2, α_2_δ_1_, iNOS, CD11b, Iba-1, GFAP, CD68, arginase-1, TNFα, and IL-1β in the ipsilateral spinal dorsal horn of mice with SNI treated with vehicle, gabapentin alone, or gabapentin combined with SnPP. The increased expression of HO-1 and decreased expressions of iNOS, Iba-1, TNF-α, and GFAP were detected in mice treated with pregabalin alone. The expressions of HO-1, iNOS, and GFAP were prevented by coadministration of SnPP. The decreased expressions of Iba-1 and TNF-α were not altered by coadministration of SnPP.

## 4. Discussion

In this study, we demonstrated that (1) SNI induced persistent mechanical allodynia, (2) an HO-1 inducer (CoPP) or CORM-2 alleviated mechanical allodynia induced by SNI, (3) the HO-1 inhibitor SnPP did not have antiallodynic effects during SNI-induced mechanical allodynia, (4) CoPP or CORM-2 enhanced the antiallodynic effects of gabapentinoids (pregabalin or gabapentin) during SNI-induced mechanical allodynia, (5) SnPP prevented the antiallodynic effects of gabapentinoids (pregabalin or gabapentin) during SNI-induced mechanical allodynia, (6) CoPP or CORM-2 increased HO-1 and Ca^2+^ channel α_2_δ_1_ subunit gene expression in the ipsilateral spinal dorsal horn in mice with SNI, (7) CoPP or CORM-2 decreased the gene expression of microglial markers, M1 microglial marker, or TNFα in mice with SNI, (8) pregabalin increased the gene expressions of HO-1 and Arg-1, decreased expressions of HO-2, iNOS, Iba-1, TNF-α, and GFAP, the altered expressions of mRNAs were prevented by coadministration of SnPP, (9) gabapentin increased the gene expression of HO-1, decreased expressions of iNOS, Iba-1, TNF-α, and GFAP, the altered expressions of HO-1, iNOS, and GFAP mRNAs were prevented by coadministration of SnPP, the decreased expressions of Iba-1 and TNF-α were not altered by coadministration of SnPP.

HO-1 induction by CoPP or CORM-2 has demonstrated anti-inflammatory and antinociceptive effects in several pain models.^6,7,16,19,32^ In line with those studies, we have shown that CoPP or CORM-2 alleviated neuropathic pain induced by SNI in mice. Hervera et al.^19^ reported that CoPP, CORM-2, or CORM-3 increases the antinociceptive effects of peripherally administered morphine during neuropathic pain induced by chronic constriction of the sciatic nerve. Conflicting findings have been obtained regarding the effects of HO-1 induction on δ-opioid agonists or cannabinoid-2 agonists during neuropathic pain.^7,19,33^ Although HO-1 induction in diabetic neuropathy enhances the antiallodynic effects of δ-opioid agonists or cannabinoid-2 agonists, HO-1 induction decreases the antiallodynic effects of δ-opioid agonists or cannabinoid-2 agonists in neuropathic pain induced by chronic constriction of the sciatic nerve. CoPP or CORM-2 has been reported to induce HO-1 in the spinal cord and reduce microglial and astrocytic activation as well as the iNOS level.^19,21,32^ In this study, CoPP or CORM-2 treatment induced HO-1 and reduced microglial markers (CD11b and Iba-1), an M1 microglial marker (CD68), and TNFα in the spinal dorsal horn. CoPP or CORM-2, however, did not change the expression of iNOS, an astrocyte marker (GFAP), or IL-1β in SNI-induced neuropathic pain. The difference between our study and previous reports may be due to the different doses of CoPP/CORM-2 (3 mg/kg in this study, 10 mg/kg or repeated doses in previous studies) or different pain models. TNFα is known to be involved in nerve injury or neuropathic pain.^37,40,42^ Reducing TNFα or using TNFα siRNA or soluble TNF receptor in the spinal cord alleviates gp120-induced HIV-related neuropathic pain.^42^ Microglia-derived TNFα induces prostaglandin I2 synthase expression in spinal endothelial cells, and endothelial prostaglandin I2 plays a critical role in neuropathic pain through neuronal prostaglandin I2 receptor.^22^ Because M2 microglial marker gene expression was not altered by CoPP or CORM-2 treatment, M2 microglia do not seem to be involved in the antiallodynic effects of HO-1 induction. Our results indicate that CoPP or CORM-2 treatment exerts antiallodynic effects through reductions in M1 microglia and the proinflammatory cytokine TNFα. CoPP or CORM-2 treatment required 4 hours to exert antinociceptive effects, and the effects were not dose-dependent. These results support the hypothesis that CoPP or CORM-2 treatment exerts antiallodynic effects through reductions in M1 microglia. In contrast to the microglial polarization to the M2 phenotype seen in the infant spinal cord, microglial polarization immediately shifts to the M1 phenotype in adult nerve injury.^15^ Spared nerve injury has been shown to increase M1 microglia in the central nervous system.^41^ The fact that inhibition of microglial activity by minocycline reversed SNI-induced mechanical allodynia further supports the hypothesis that CoPP or CORM-2 treatment exerts antiallodynic effects through reductions in M1 microglia.

We have shown that CoPP or CORM-2 treatment significantly increases the α_2_δ_1_ subunit of voltage-gated Ca^2+^ channels. Nerve injury increases expression of the α_2_δ_1_ subunit.^4^ Genetically increased expression of the α_2_δ_1_ subunit reportedly induces mechanical allodynia.^29,43^ The increased expression of the α_2_δ_1_ subunit may hamper the CoPP- or CORM-2–induced analgesic effects. We suggest that when α_2_δ_1_ is blocked, the limitation to CoPP and CORM effectiveness may be removed. The maximum analgesic effects of CoPP and CORM may be added to other antiallodynic effects of gabapentinoids. Although gabapentinoids are believed to exert antinociceptive effects mainly through the Ca^2+^ channel α_2_δ_1_ subunit, serotonin- or noradrenaline-mediated descending modulation might be involved in the effects of gabapentinoids.^9,24^ Previous studies have shown that gabapentin activates the interleukin-10/HO-1 signaling pathway and enhances morphine's antinociceptive effects.^3,27^ HO-1 inhibitors partially block the effect of gabapentin on morphine. In our study, the HO-1 inhibitor SnPP prevented the antiallodynic effects of high doses of gabapentinoids. Pregabalin significantly increased expressions of HO-1 and Arg-1 and decreased expressions of HO-2, iNOS, Iba-1, TNF-α, and GFAP. The altered expressions of mRNAs were prevented by coadministration of SnPP. The results indicate that pregabalin induces M2 microglia and suppresses glial activation, iNOS, and HO-2 through HO-1 induction. Hervera et al.^20^ have reported that HO-1 induction suppresses glial activation through iNOS inhibition during neuropathic pain. Li and Clark have reported that spinal HO-2 induction is involved in mechanical allodynia during inflammatory or neuropathic pain.^30,31^ Our results are in line with these reports. Although gabapentin also increased expression of HO-1 and decreased expressions of iNOS, Iba-1, TNF-α, and GFAP, the expression of HO-2 or Arg-1 was not changed by gabapentin. Coadministration of SnPP prevented the altered expressions of HO-1, iNOS, and GFAP. Coadministration of SnPP, however, did not prevent the altered expressions of Iba-1 and TNF-α. These results indicate that pregabalin and gabapentin may have different effects on the glial cell activation induced by SNI. These results also indicate that HO-1 signaling plays an important role in the antinociceptive effects of gabapentinoids.

In conclusion, this study suggests that HO-1 plays crucial roles in the antiallodynic effects of gabapentinoids. Gabapentinoids attenuate the glial activation induced by SNI, and some of these effects are mediated by HO-1.

## Disclosures

The authors have no conflict of interest to declare.

This work was supported by JSPS KAKENHI Grant Number JP 17K16740 to K. Godai.

Part of this study was presented at the 65th annual meeting of the Japanese Society of Anesthesiologists (2018, Yokohama, Japan).
